# Comparison of 2-Hydroxyglutarate Detection With sLASER and MEGA-sLASER at 7T

**DOI:** 10.3389/fneur.2021.718423

**Published:** 2021-09-07

**Authors:** Zahra Shams, Wybe J. M. van der Kemp, Uzay Emir, Jan Willem Dankbaar, Tom J. Snijders, Filip Y. F. de Vos, Dennis W. J. Klomp, Jannie P. Wijnen, Evita C. Wiegers

**Affiliations:** ^1^Department of Radiology, University Medical Center Utrecht, Utrecht, Netherlands; ^2^School of Health Sciences, Purdue University, West Lafayette, IN, United States; ^3^Department of Neurology & Neurosurgery, University Medical Center Utrecht/UMC Utrecht Brain Center, Utrecht, Netherlands; ^4^Department of Medical Oncology, University Medical Center Utrecht, Utrecht, Netherlands

**Keywords:** 2-hydroxyglutarate, ultra-high field, SV MRS, MEGA-sLASER, sLASER, J-difference editing

## Abstract

The onco-metabolite 2-hydroxyglutarate (2HG), a biomarker of IDH-mutant gliomas, can be detected with ^1^H MR spectroscopy (^1^H-MRS). Recent studies showed measurements of 2HG at 7T with substantial gain in signal to noise ratio (SNR) and spectral resolution, offering higher specificity and sensitivity for 2HG detection. In this study, we assessed the sensitivity of semi-localized by adiabatic selective refocusing (sLASER) and J-difference MEsher-GArwood-semi-LASER (MEGA-sLASER) for 2HG detection at 7T. We performed spectral editing at long TE using a TE-optimized sLASER sequence (110 ms) and J-difference spectroscopy using MEGA-sLASER (*TE* = 74ms) in phantoms with different 2HG concentrations to assess the sensitivity of 2HG detection. The robustness of the methods against B_0_ inhomogeneity was investigated. Moreover, the performance of these two techniques was evaluated in four patients with IDH1-mutated glioma. In contrary to MEGA-sLASER, sLASER was able to detect 2HG concentration as low as 0.5 mM. In case of a composite phantom containing 2HG with overlapping metabolites, MEGA-sLASER provided a clean 2HG signal with higher fitting reliability (lower %CRLB). The results demonstrate that sLASER is more robust against field inhomogeneities and experimental or motion-related artifacts which promotes to adopt sLASER in clinical implementations.

## Introduction

Mutations in IDH1 and IDH2 encoding genes result in the accumulation of 2-hydroxyglutarate (2HG) in glial brain tumor cells (1, 2). 2HG can therefore serve as a biomarker for IDH mutated gliomas (3). IDH mutations are highly diagnostic for diffuse gliomas and form an integral part of the contemporary WHO classification of brain tumors (4). It has been shown that patients with IDH1 and IDH2 mutated gliomas have better survival rates than patients with IDH wild type tumors (1, 5). Recent studies demonstrated that IDH mutations in glioma predict the response to chemotherapy in anaplastic glioma (6). Hence, the detection of 2HG in IDH-mutated gliomas can aid in the diagnosis and treatment planning of glioma patients. This can be achieved non-invasively with MR spectroscopy (MRS), provided that dedicated sequences are being used.

The signal of 2HG at 2.25 ppm (Hγ) in the MR spectrum overlaps with signals of glutamate, glutamine and γ-aminobutyric acid (GABA). Consequently, the specificity of 2HG detection is low when using MRS sequences with a short echo time (7, 8). To mitigate this problem, spectral editing at long echo time and J-difference spectroscopy became of interest (9, 10). The pioneers of these approaches at 3T were Choi et al. They optimized the point-resolved spectroscopy (PRESS) sequence for 2HG detection at an echo time (TE) of 97 ms (9), with reasonable sensitivity and specificity. To overcome overlapping of metabolite signals in the frequency range of 2HG's Hγ signal, MEsher-GArwood-PRESS (MEGA-PRESS) sequence (TE = 68 ms) was introduced. This sequence detects the H_α_ signal at 4.02 ppm with a more accurate quantification of 2HG levels compared to PRESS 97 ms (11). Andronesi et al. (10) designed a J-difference editing sequence specific to 2HG detection (*TE* = 75 ms), based on the LASER (localization by adiabatic selective refocusing) technique to alleviate the chemical shift displacement error compared to the PRESS sequence.

Increased spectral resolution at ultra-high field strengths (≥7T) allows a more reliable detection of overlapping metabolite signals such as Glutamate (Glu) and Glutamine (Gln) (12). Together with the increased signal to noise ratio at ultra-high field strengths, subtle changes in metabolite levels can be detected which leads to highly improved precision and quantification accuracy (13). This can be beneficial for 2HG detection, since 2HG levels can be lower than 5 mM in tumor tissue (8, 11, 14). At 7T, long echo time (110 ms) semi-localized by adiabatic selective refocusing (sLASER) sequence for 2HG detection was proposed and proved to outperform its PRESS counterpart because of less chemical shift displacement error in addition to less sensitivity to ${\text{B}}_{1}^{+}$ inhomogeneities (15, 16). Based on quantum-mechanical simulations, the optimal echo time and inter-pulse delays in this scheme were obtained, which resulted in a fully absorptive negative lineshape for 2HG at 2.25 ppm. This substantially improved discrimination between J-coupled spin resonances of 2HG and Glu, Gln, Glutathione (GSH), and GABA compared to long-TE (110 ms) sLASER at 3T (17). Moreover, 2HG-optimized echo time of 110 ms resulted in significantly higher Hγ signal at 2.25 ppm in comparison to data acquired with a short TE of 36 ms (15).

A “cleaner” signal for 2HG may be obtained when using J-difference editing, even more at ultra-high field as the chemical shift dispersion is larger, and consequently co-editing of other metabolites is reduced. Also, much narrower spectral selectivity can be obtained with the same editing pulse bandwidth as used at 3T. Utilizing the unique resonance at 4.02 ppm of 2HG in J-difference editing could make this technique very robust and specific for 2HG detection. However, the main drawback of performing editing at 7T is that the water suppression is more challenging, which may affect the 2HG resonance at 4.02 ppm.

So far, long echo time acquisitions have been used as the most robust method for 2HG detection at 7T, while the feasibility and performance of edited MRS remained unclear. In this study, we compare long-TE sLASER and J-difference editing MEGA-sLASER acquisitions for 2HG detections at 7T regarding robustness and reliability. Furthermore, the resulting spectra from four patients with IDH-mutant glial tumors are shown as practical examples of applying these methods at 7T.

## Materials and Methods

### Phantom Preparation

The sensitivity of the two methods in 2HG detection was assessed in five spherical phantoms with a diameter of 4 cm. Four phantoms contained 2HG (DL-α-Hydroxyglutaric acid disodium salt, Sigma Aldrich) at different concentrations and glycine (Gly) as a reference peak where the concentrations of 2HG/Gly (mM/mM) were 0.5/5, 1/10, 1.6/10, 5.8/10. The fifth phantom contained 5 mM 2HG, 1 mM gamma-aminobutyric acid (GABA), 5 mM glutamate (Glu), 5 mM glutamine (Gln), 10 mM creatine (Cr), 10 mM n-acetyl aspartic acid (NAA), and 7 mM myo-inositol (mIn). We added PBS (phosphate-buffered saline) solution to the phantoms as a buffer solution. The pH was adjusted to 7.2 by adding small drops of sodium hydroxide whilst measuring pH with a pH-meter (SevenCompact S210 from Mettler Toledo).

### Acquisitions

**Figure 1:**
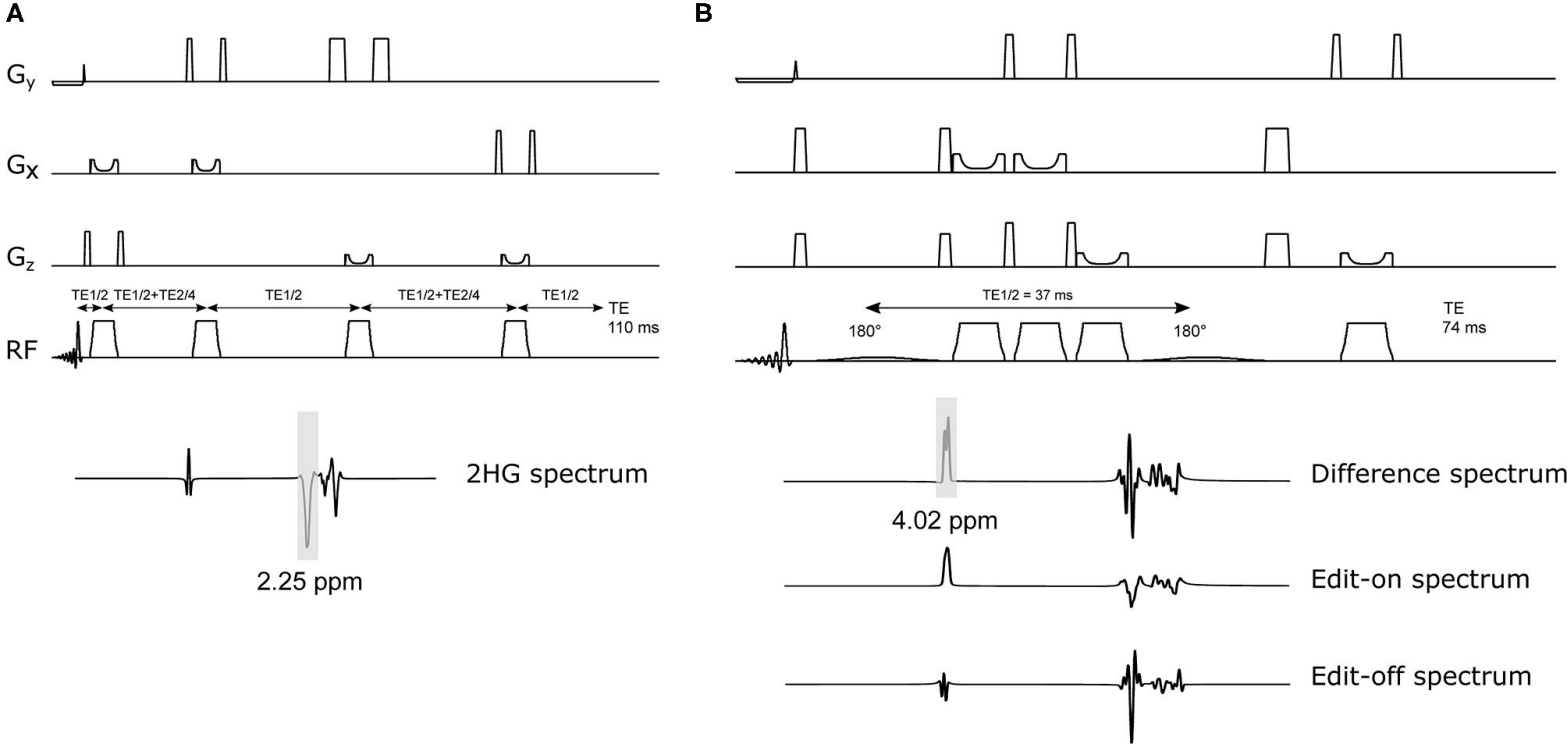
Schematic of 2HG-optimized sLASER and MEGA-sLASER with their corresponding simulated 2HG spectra. **(A)** sLASER sequence with 2HG-optimized TE of 110 ms (TE1, TE2, and TE3 = 11, 65, and 34 ms). The bandwidth of the FOCI pulses in both sequences is ~11 kHz (10.78 kHz). **(B)** MEGA-sLASER sequence with two 180° frequency selective pulses centered at 2HG β frequency (1.9 ppm) and two pairs of FOCI pulses for adiabatic localization. The frequency selective pulses are Gaussian (truncated at 10%) with bandwidth = 88Hz. The difference spectrum is obtained by the subtraction of Edit-on and Edit-off spectra.

MR experiments were performed on a 7T MR scanner (Philips, Achieva, Best, NL) equipped with a 32-channel receive-only and 8-channel transmit coil (Nova Medical, Inc., Burlington, MA, USA). We performed sLASER measurements at TE = 110 ms and the delays between the frequency offset corrected inversion (FOCI) refocusing pulses were tuned as in Emir et al. (15) to achieve optimized J-coupling patterns for 2HG (Figure 1A). Spectral editing with MEGA-sLASER, with a TE = 74 ms adapted from Andreychenko et al. (18), was performed using two 180° frequency selective Gaussian pulses with a bandwidth of 88 Hz which were tuned symmetrically from the water resonance at 1.9 and 7.5 ppm, in the odd and even acquisitions, respectively (Figure 1B).

### Phantom Experiments

The offset frequencies of the Gaussian pulses were shifted 0.12 ppm from the patient values because of the lower temperature of the phantom (room temperature). The carrier frequency of the FOCI pulses for sLASER and MEGA-sLASER were 2.7 and 3.0 ppm, respectively. The following acquisition parameters were kept the same for all experiments: number of signal averages (NSAs) was 128 for all phantoms and 256 for the one with lowest concentration of 2HG. TR (5 s); spectral width (6 kHz); voxel size (8 cm^3^ isotropic); transverse voxel orientation, VAPOR water suppression (19); 16 steps of phase cycling; and number of complex points (np) 2048. B_0_ was optimized for the selected volumes by FASTMAP (20) with up to 2nd order shim terms (PB-2nd) using the MRS voxel as a target volume. In additional experiments, we assessed the performance of the two methods in less optimal shimming conditions, as we expected in *in-vivo* experiments. The local B_0_ field in the voxel was perturbed by changing the z component of the shim gradients up to 0.045 mT/m.

### Patient Study

The study was approved by the local ethical committee. We obtained informed consent from four patients with IDH1-mutated glioma who then participated in this study: patient 1 and 2 with diffuse astrocytoma, WHO grade II, patient 3 with anaplastic astrocytoma, WHO grade III, and patient 4 with diffuse astrocytoma, WHO grade II, that developed to grade IV at time of progression. All IDH-mutations were confirmed by a certified neuropathologist by means of next-generation sequencing. These patients had received different types of treatments i.e., chemotherapy, proton-beam irradiation and photon radiotherapy. An overview of clinical characteristics, including exact IDH1-mutation as well as previous and ongoing treatments at time of the MRS are given in Supplementary Table 1. The location of the MRS voxel within the tumor was appointed by an experienced neuroradiologist (JWD) on a T2-weighted FLAIR image (3D TSE sequence with 1 mm isotropic resolution). MRS measurements were performed with the same acquisition parameters as the phantom study. Patient 4 had titanium surgical material in his skull, therefore we had to lengthen the TR to 6 s to remain within SAR (specific absorption rate) limits. NSAs for sLASER and MEGA-sLASER were 64 and 128, and for patient 1 were 32 and 64, respectively. 2HG spectra were acquired from voxel sizes of 8 cm^3^ (patient 1), 9.6 cm^3^ (patient 2), 4.98 cm^3^ (patient 3), and 8 cm^3^ (patient 4). No additional outer volume suppression (OVS) was used except for patient 1.

### Data Processing and Analysis

The data were coil combined (21), frequency and phase aligned with the FID-A toolbox (22). For MEGA-sLASER, the on and off spectra were subtracted after phase and frequency alignment followed by averaging. All the spectra were analyzed using LCModel (23). Spectral simulation was done using Vespa library of GAMMA (24) to create the basis sets for LCModel analysis. The concentration of 2HG in the 2HG-Gly phantoms was estimated using the water spectrum as a reference. The water concentration was assumed to be 55.6 M (bulk water) in the phantom. Estimated 2HG and Gly concentrations were corrected for T2 relaxation [T2 (Gly) = 1,500 ms and T2 (2HG) = 650 ms] (16). The effect of T1 relaxation rate was not taken into account. The metabolites included in the basis set for the sLASER experiment were: 2-HG, alanine (Ala), ascorbate (Asc), aspartate (Asp), creatine (Cr), GABA, Gln, Glu, glycine (Gly), myo-Inositol, Lactate (Lac), N-acetylaspartylglutamate (NAAG), phosphocholine (PCho), phosphocreatine (PCr), phosphoethanolamine (PE), scyllo-inositol (sIns), taurine (Tau), glucose (Glc), glycerophosphocholine (GPC), and GSH. The basis set for MEGA-sLASER experiment included 2HG, GABA, Glu, Gln, GSH, NAA, and NAAG. We set the DKNTMN parameter of LCModel to 0.2 to make the spline baseline flexible enough to automatically account for highly variable macromolecule and lipid signals. For sLASER with long TE of 110 ms, the macromolecules contribution can be neglected (25).

For the 2HG-Gly phantoms, the edited spectra only contained signals from 2HG.The relative 2HG and Gly concentration in these phantoms was calculated by deriving the concentration of Gly from the edit-off spectra (spectra with editing pulses tuned on 7.5 ppm).

## Results

**Figure 2:**
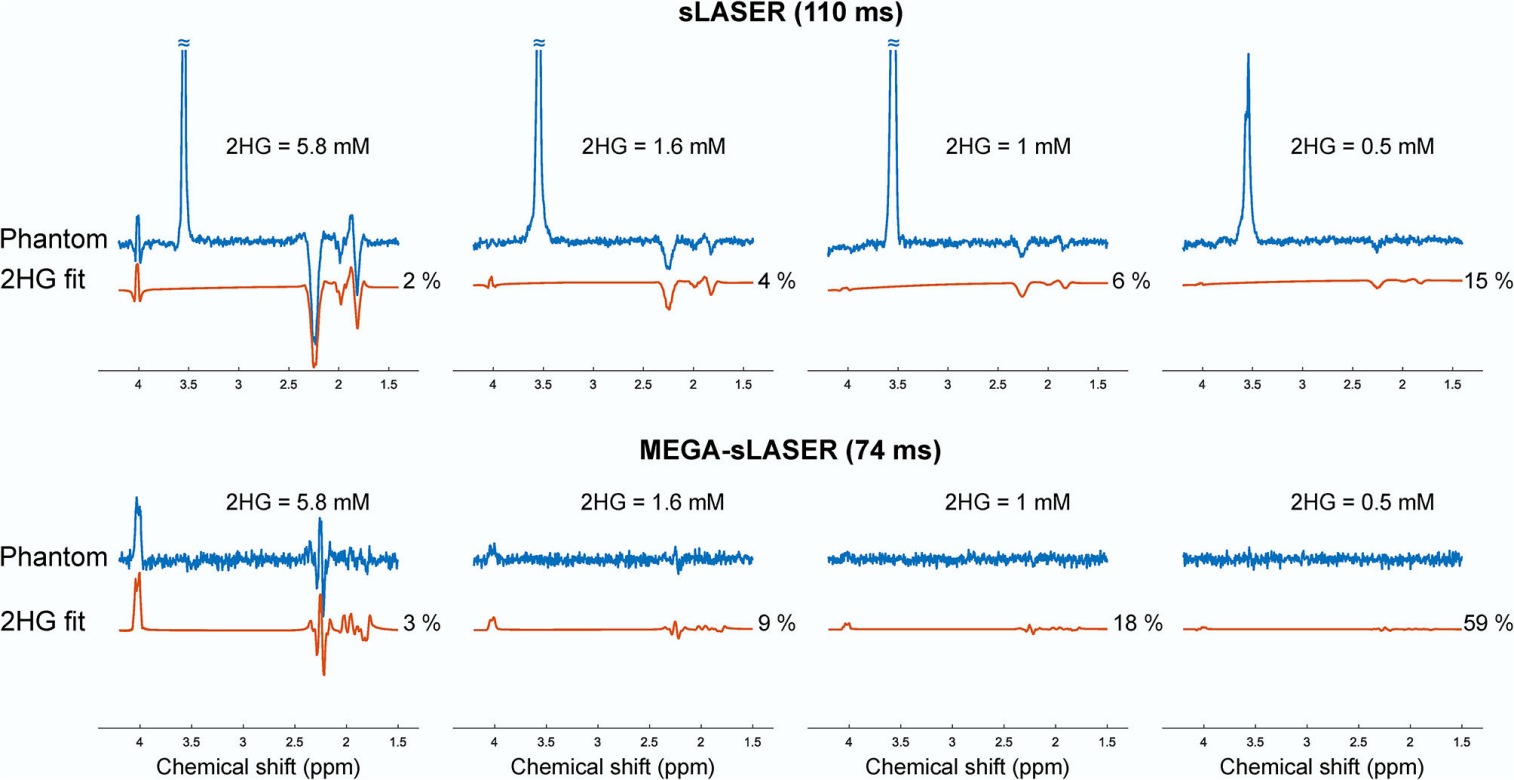
Spectra and the 2HG fits of the four phantoms containing 2HG (5.8, 1.6, 1, and 0.5 mM) and glycine (10, 10, 10, and 5 mM) from sLASER TE 110 ms (top) with MEGA-sLASER TE 74 ms (bottom) experiments. The spectra in each row were scaled to standard deviation of the noise.

**Figure 3:**
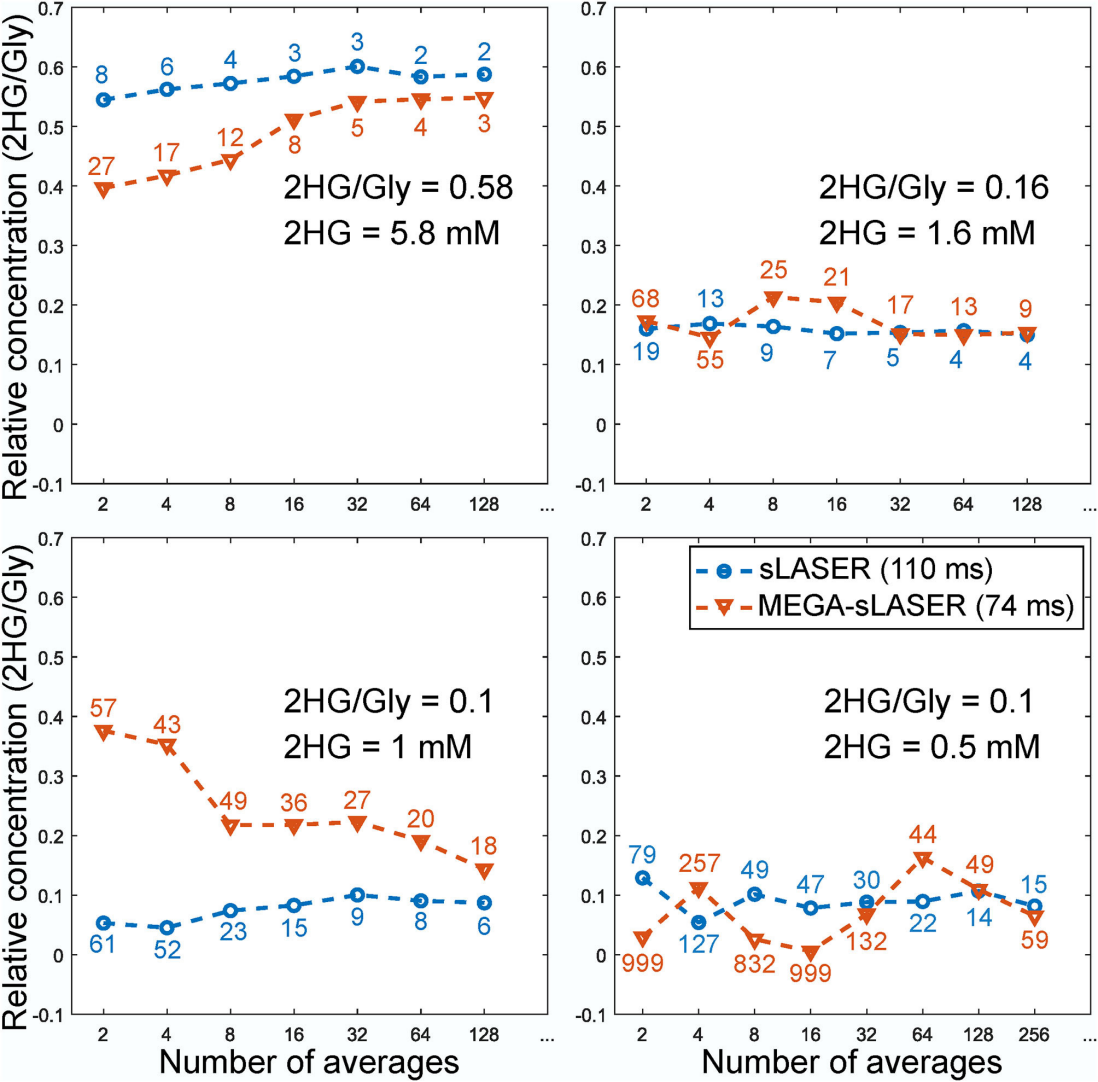
Relative concentration of 2HG to glycine in the phantoms as a function of number of averages. The numbers correspond to the CRLBs of the 2HG fit. The concentration of Gly for MEGA-sLASER measurements was derived from the edit-off spectra.

Figure 2 shows the LCModel fits in 2HG-Gly phantoms with corresponding CRLBs for both sLASER and MEGA-sLASER acquisitions. Using J-difference editing, the multiplet at 4.02 ppm is resolved in the spectrum while removing the signal contribution from Gly after the subtraction of two subsequent acquisitions, with and without the frequency selective pulse. In both techniques, 2HG can be detected with a CRLB (%) of <20% except for the phantom with lowest 2HG concentration [2HG/Gly (mM) = 0.5/5] where MEGA-sLASER failed according to the CRLB of the fit 59%. Using sLASER, we were able to detect 2HG concentration of as low as 0.5 mM (CRLB = 30%) with at least 32 number of signal averages. sLASER and MEGA-sLASER were able to reproduce the real 2HG-Gly concentration ratio of 0.58 (5.8/10 mM) in the corresponding phantom with the errors of 1.3 and 5.4%, respectively. For the phantom with real 2HG-Gly relative concentration of 0.16 (1.6/10 mM), the errors produced by sLASER and MEGA-sLASER were 1.28 and 0.6%, respectively. The mean errors of the relative concentration for the other two phantoms were 9.9% for sLASER and 26.3% (2HG overestimation in both phantoms) for MEGA-sLASER. The 2HG to Gly ratio (After T2 correction) is plotted as a function of the number of signal averages in Figure 3. Only considering the NSAs where the 2HG %CRLB was lower than 30%, MEGA-sLASER resulted in overestimation of 2HG/Gly in the phantom with 1 mM 2HG.

**Figure 4:**
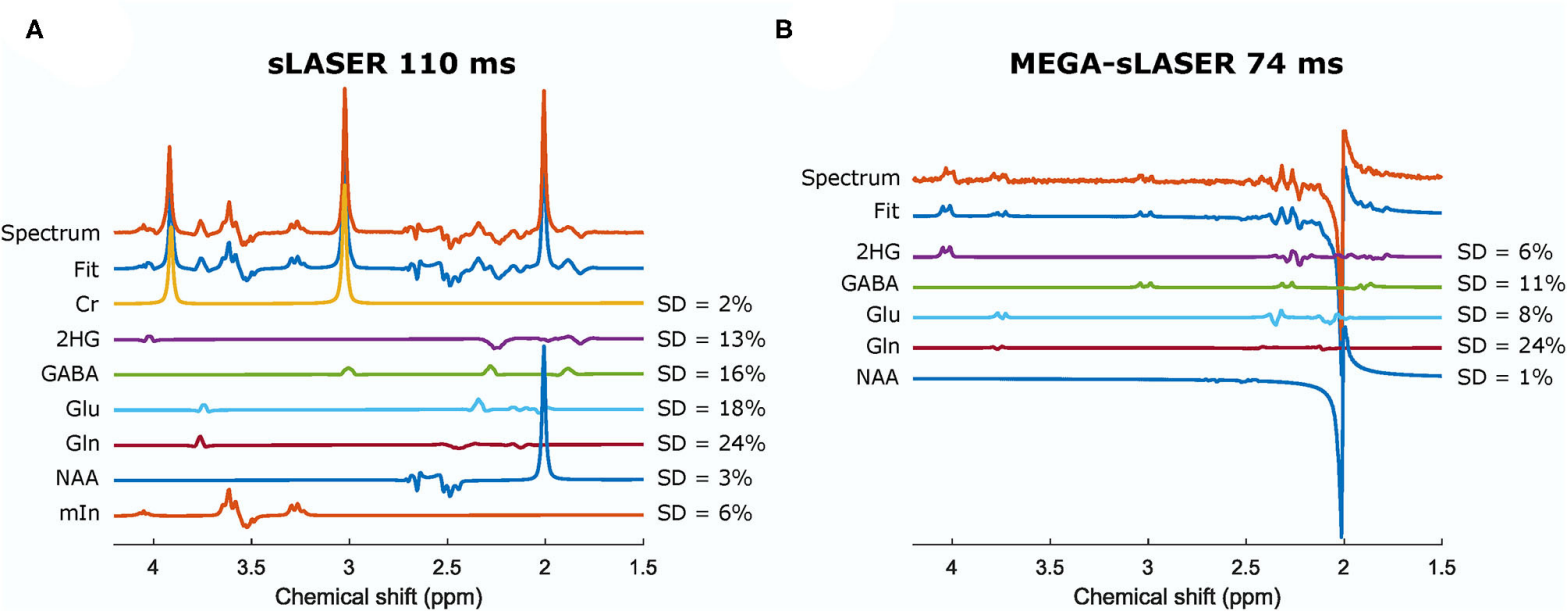
Spectra and the fits from a phantom containing 2HG, Cr, NAA, Glu, Gln, GABA, and mIn acquired from sLASER TE = 110 ms **(A)** and MEGA-sLASER TE = 74 ms **(B)**. The relative concentrations (/NAA) can be found in Table 2. Cr, creatine; NAA, N-acetylaspartate; Glu, glutamate; Gln, glutamine; GABA, γ-aminobutyric acid; mIn, myo-inositol.

**Figure 5:**
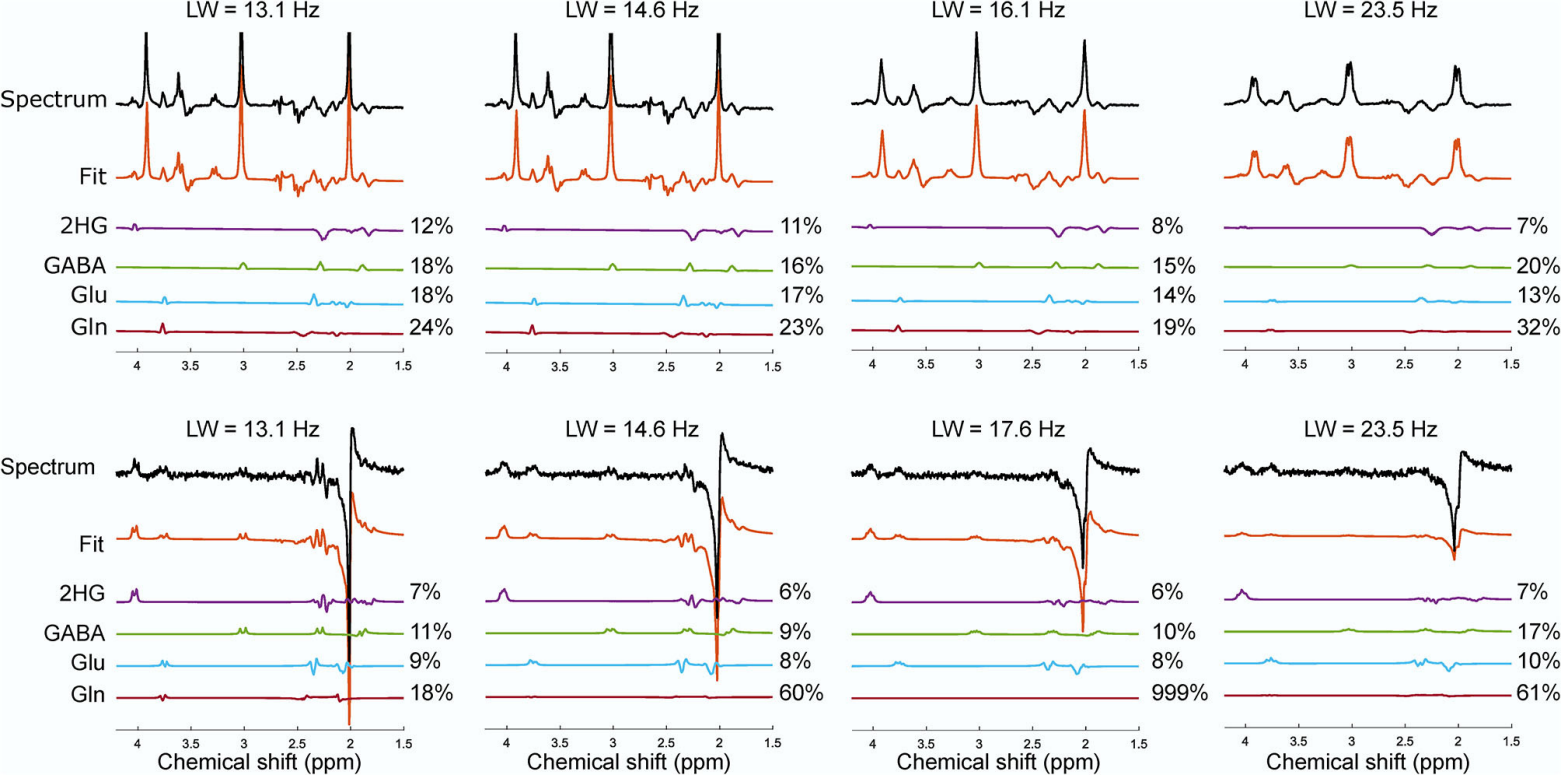
The effect of the increase in linewidth on the reported CRLB (%) from the phantom with NAA, Cr, 2HG, Glu, Gln, GABA, myo-inositol for sLASER (top row) and MEGA-sLASER (bottom row). The spectra with their corresponding fit for 2HG and other overlapping metabolites of GABA, Glu and Gln from sLASER and MEGA-sLASER measurements at different linewidth are shown. Number of averages for sLASER and MEGA-sLASER were 32 and 64, respectively. The spectra were scaled to the standard deviation of the noise.

In the presence of other overlapping metabolites, MEGA-sLASER resulted in more precise detection of 2HG with relative concentration (/NAA) of 0.43 (CRLB = 6%) compared to 0.36 (CRLB = 13%) with sLASER as shown in Figure 4 [the true relative concentration (/NAA) = 0.5]. In addition, the GABA CRLB was lower (CRLB = 11%) with MEGA-sLASER compared to sLASER (CRLB = 16%). Tables 1, 2 summarize the effect of metabolite signal overlap on CRLB (%) and concentrations (/NAA) by removing the overlapping metabolites from the basis set. Excluding GABA from the basis set had negligible or even no influence on detecting 2HG using MEGA-sLASER, while the %CRLB for 2HG decreased (from 13 to 5%) after GABA's removal from the basis set in sLASER technique. Removing Glu and Gln from the basis set had almost no influence on quantification of 2HG using sLASER (Table 2). While the reliability of 2HG fit (%CRLB) was affected by the linewidth in the sLASER sequence, the MEGA-sLASER sequence exhibited the same %CRLB of the metabolites in MR spectra with increasing linewidth (Figure 5). The reported linewidth is the water linewidth reported by LCModel.

**Table 1:** CRLB (%) of overlapping metabolites.

		**CRLB (%)**				
		**2HG**	**GABA**	**Glu+Gln**	**Glu**	**Gln**
Without 2HG	MEGA-sLASER		13	12	10	32
	sLASER		20	14	17	29
Without GABA	MEGA-sLASER	6		8	8	22
	sLASER	5		14	21	23
Without Glu-Gln	MEGA-sLASER	7	10			
	sLASER	14	18			
All metabolites	MEGA-sLASER	6	11	9	8	24
	sLASER	13	15	13	18	24

*Overlapping metabolites such as 2HG, GABA, Glu, and Gln were removed from the basis set to examine the influence on the resulting fit. Gray shaded areas represent the excluded metabolites*.

**Table 2:** The relative concentrations (/NAA) obtained from MEGA-sLASER and sLASER when excluding the overlapping metabolites from the basis set.

		**Concentration (/NAA)**				
		**2HG**	**GABA**	**Glu+Gln**	**Glu**	**Gln**
Without 2HG	MEGA-sLASER		0.09	0.42	0.28	0.15
	sLASER		0.13	0.72	0.41	0.31
Without GABA	MEGA-sLASER	0.42		0.52	0.34	0.18
	sLASER	0.33		0.73	0.33	0.4
Without Glu-Gln	MEGA-sLASER	0.39	0.11			
	sLASER	0.36	0.15			
All metabolites	MEGA-sLASER	0.43	0.09	0.47	0.31	0.15
	sLASER	0.36	0.16	0.73	0.37	0.36

*True concentrations are: 2HG/NAA = 0.5, GABA/NAA = 0.1, Glu/NAA = 0.5, Gln/NAA = 0.5. Gray shaded areas represent the excluded metabolites*.

**Figure 6:**
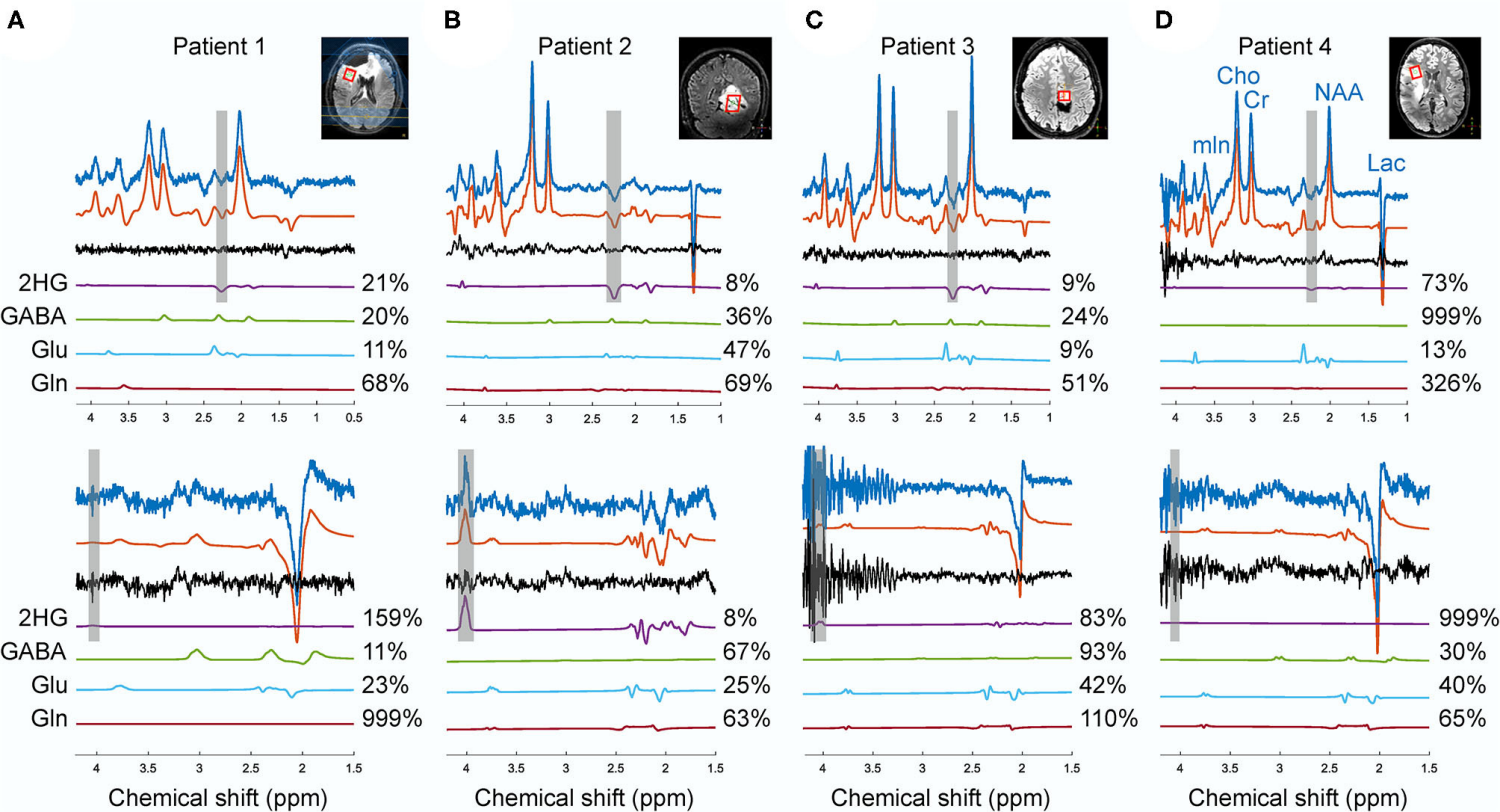
The spectra from sLASER (upper spectra) and MEGA-sLASER (lower spectra) acquired in patients 1 **(A)**, 2 **(B)**, 3 **(C)**, 4 **(D)** with histologically confirmed IDH1-mutated glioma. The frequency ranges of 2HG γ (2.25 ppm) and α (4.02 ppm) signals are shaded in gray. The voxel sizes for patients 1, 2, 3, and 4 were 8, 9.6, 4.98, and 8 cm^3^, respectively.

Figure 6 shows the spectra from the four patients that participated in this study. The choline linewidth for Patient 1, 2, 3, and 4 were 29.13, 11.49, 12.45, and 15.71 Hz, respectively. For these patients, partial volume of the tumor in the MRS voxel was estimated by a neuroradiologist (JWD) as the percentage of the MRS voxel filled with the FLAIR abnormality that were 90, 90, 80, and 80% with estimated tumor likelihood (the possibility of the abnormal tissue in the MRS voxel being tumorous) of 25, 100, 75, and 50%, respectively. In patient 1, with low probability of the MRS voxel being filled with genuine tumor, 2HG was detected by sLASER (%CRLB = 27) while no 2HG was detected with MEGA-sLASER (%CRLB = 159). In patient 2 with high tumor likelihood and high partial volume of tumor, both methods perform well in 2HG detection. By excluding 2HG from the sLASER basis set, a residual signal is seen at 2.25 ppm from the fit although CRLB of GABA improved a bit. The peak at around 4.02 ppm in the edited spectrum can be purely assigned to 2HG. In patient 3, the editing method failed. For the chosen voxel location, the spoilers were not strong enough, leading to large spurious echoes around the 2HG frequency at 4.02 ppm. When excluding 2HG from the basis set, the fit resulted in residuals at 2HG resonance frequency of 2.25 ppm (Supplementary Figure 1A). The correlation coefficients between 2HG and either GABA, Glu or Gln reported by LCModel for this case were 0.26, −0.2, and 0.22 respectively, meaning that the 2HG signal did not exhibit significant correlation with those metabolites. In patient 4 no 2HG was detected with either MEGA-sLASER or sLASER. Again, by removing 2HG from the basis set, no considerable change in the residual at 2.25 can be observed (Supplementary Figure 1B).

## Discussion and Conclusion

In this study, we compared sLASER and MEGA-sLASER for detecting 2HG at 7T *in vitro* and in patients. The sensitivity of the two methods for 2HG detection was assessed with four phantoms containing 2HG at different concentrations ranging between 0.5 and 5.8 mM. At low SNR and low levels of 2HG (e.g., 1 mM), the 2HG concentration using MEGA-sLASER was overestimated (Figure 3). sLASER appeared to be more sensitive to 2HG detection at lower concentrations (0.5 mM with CRLB of 15%) where MEGA-sLASER failed in detecting 2HG according to the reported CRLB of 59%. Also, according to 2HG-Gly ratio, the LCModel fits of sLASER were more consistent across the number of NSAs. It could be that the LCModel fit of 2HG in the edited spectra is suboptimal because of the absence of a prominent Gly peak. This situation could occur in fitting of edited MR spectra of tumors with very low NAA levels where subtraction of the spectra leads to only 2HG signals with some other co-edited metabolites such as GABA and Glu. We showed an example of such a spectrum in Figure 6 (patient 2).

The effect of linewidth on 2HG detection was evaluated by gradually increasing the z component of the shim and analyzing the changes in CRLB of the 2HG fit. When the linewidth was larger (a change from 13.1 to 14.6 Hz), almost all the CRLBs for 2HG (12 and 11%) and other overlapping metabolites remained the same in the sLASER method. But the linewidth of 16.1 Hz led to a decrease in 2HG CRLB (8%) and a change in CRLBs of other metabolites. That means a linewidth of larger than 16 Hz will cause some signal stealing between 2HG and the overlapping metabolites in the sLASER sequence. On the other hand, broader linewidth did not have a noticeable impact on the fit reliability (%CRLB) for the MEGA technique where the edited signal at 4.02 ppm is uniquely generated via the J-couplings of 2HG. Despite the optimized interpulse delay aiming at a distinct lineshape for 2HG, the remaining overlapping resonances from GABA, Glu and Gln, combined with poorer shim conditions in brain tumor tissue, may be more problematic than demonstrated here in phantoms. Nevertheless, *in vivo* MEGA-sLASER might be more sensitive to B_0_ inhomogenities for two reasons; firstly, narrow-band frequency selective pulses tuned on specific frequencies would not perform optimally under strong inhomogeneous B_0_ conditions, which will consequently affect editing efficiency even though CRLB reports low numbers. Secondly, efficiency of water suppression will be also reduced, which ultimately leads to the 2HG resonance at 4.02 ppm being obscured by water signal (26, 27). The acquisition in patient 1, post-surgery and treated with chemotherapy, is an example of such a situation. The broad linewidth and its consequent neighboring signals contamination (2HG signal stealing from Glu and GABA) might be the reason for the low 2HG CRLB (27%) for sLASER; the same as observed in the phantom with linewidth of 23 Hz.

In addition to the phantom study, we showed some practical examples to assess the performance of these methods in patients. The four patients participated in this study had been under various treatments. Therefore, the presence and level of 2HG at the time of the scan was ambiguous. However, these cases demonstrate the practicality of each method for evaluating the performance of these two techniques in terms of robustness in patients. As discussed above, reliable detectability of 2HG depends on the linewidth. In our patient study, except for patient 1, the quality of the B_0_ shimming was acceptable [according to Juchem et al. (28)] with the mean *in vivo* choline linewidth of 13.22±2.21 Hz. In terms of spectral quality, sLASER was more robust across the patients with various voxel locations and sizes. For patient 2 (no surgery), both methods succeeded in detection of 2HG. Most probably, suboptimal B_0_ shimming outside the voxel adjacent to the post-surgery cavity compromised water suppression and led to generation of spurious echoes in the MEGA-sLASER spectrum for patient 3 (Figure 6C).

All the patients had undergone surgery, either biopsy or subtotal resection, before their MR scan and had been receiving chemo- and/or radiotherapy when participating in this study. As this study did not focus on the effect of treatment, we did not evaluate the 2HG concentration in relation to treatment and therapy. Therefore, we compared the two methods based on the 2HG CRLB reported by LCModel rather than the 2HG levels.

To have comparable SNR for the sLASER and MEGA-sLASER, the MEGA-editing measurements in patients were acquired with twice as many NSA and therefore a longer scan time for MEGA-sLASER. Being based on the subtraction of two spectra, editing techniques are susceptible to motion and scanner frequency drift which can result in underestimation of 2HG signal. Real-time motion and B_0_ correction may help to achieve more stability against motion and scanner-induced artifacts (29). In addition to a higher sensitivity of edited MRS to temporal frequency drift or B_0_ inhomogeneities, frequency selective Gaussian pulses used as editing pulses are sensitive to changes in ${\text{B}}_{1}^{+}$ field. Gaussian frequency selective pulses had a very narrow bandwidth which resulted in dependence of refocusing efficiency to ${\text{B}}_{1}^{+}$ (Supplementary Figure 2). It has been shown for GABA editing that the integrated signal of the edited multiplet decreased by a factor of approximately 15 when the ${\text{B}}_{1}^{+}$ is 70 or 130% compared to 100% (29), which could cause a false negative even with a low CRLB. Therefore, due to their ${\text{B}}_{1}^{+}$-insensitivity, asymmetric adiabatic editing pulses are favorable at 7T (29). However, adiabatic editing pulses will increase the RF deposition and therefore an even longer TR and acquisition time would be required.

Metabolic imaging of many more glioma-associated oncometabolites than 2HG has been explored recently (30, 31). Ultra high field provides robust molecular feature extraction *in vivo* (32) that facilitates high precision non-invasive clinical biomarker imaging for diagnosis in glioma patients. This suggests a need for an MRS sequence capable of simultaneous detection of those oncometabolites with high sensitivity and specificity, and thereby excluding the use of editing sequences. The 2HG-optimized sLASER 110 ms demonstrated both distinct cystathionine and 2HG peaks in oligodendroglioma patients with IDH-mutation and 1p/19q codeletion at 7T (33).

In conclusion, the patients' results showed that the MEGA-editing method is less robust in cases of poor water suppression and B0 and B1 inhomogeneities than sLASER. Together with the finding that the fit reliability for 2HG in sLASER is similar to MEGA-sLASER promotes to choose for a sLASER implementation in clinical setting. Also, sLASER is a simpler implementation with the benefit of preserving signals from all other metabolites.

## Data Availability Statement

The raw data supporting the conclusions of this article will be made available by the authors upon personal request.

## Ethics Statement

The studies involving human participants were reviewed and approved by the Medical Ethics Review Board (METC) from the University Medical Center Utrecht. The patients provided their written informed consent prior to participation in this study.

## Author Contributions

ZS, JW, EW, UE, JD, and DK designed the study. ZS and WK made the phantoms. ZS, EW, and JW conducted the experiments. ZS analyzed the data. ZS, JW, EW, and JD were involved in data interpretation. JD was responsible for patient recruitment and assigned the lesions and performed the radiological reading. TS and FV assisted in patient recruitment and reviewed clinical data. All authors drafted the manuscript and approved the final version of the manuscript.

## Funding

This study was supported by funding from the H2020 program of the European Union; Eurostars Project *E!12449 IMAGINE!*. 

## Conflict of Interest

The authors declare that the research was conducted in the absence of any commercial or financial relationships that could be construed as a potential conflict of interest.

## Publisher's Note

All claims expressed in this article are solely those of the authors and do not necessarily represent those of their affiliated organizations, or those of the publisher, the editors and the reviewers. Any product that may be evaluated in this article, or claim that may be made by its manufacturer, is not guaranteed or endorsed by the publisher.
