# Supplementary material for: Comparison of 2-Hydroxyglutarate Detection With sLASER and MEGA-sLASER at 7T
Source: Front Neurol. 2021 Sep 7;12:718423. doi: 10.3389/fneur.2021.718423 (PMC8452903; doi:10.3389/fneur.2021.718423)
Supplement: Supplementary file 1 [file Table_1.DOCX]

Supplementary Material

Supplementary Table 1. An overview of clinical characteristics, including exact IDH1-mutation as well as previous and ongoing treatments at time of the MRS scan for all four patients

|  | **Type of IDH mutation^*^** | **Diagnosis, WHO 2016 classification** | **Treatments preceding MRI** |
| --- | --- | --- | --- |
| **Patient 1** | IDH1 c.395G>A; p.(Arg132His) | Astrocytoma, IDH-mutant, WHO grade II | Subtotal resection  Temozolomide chemotherapy |
| **Patient 2** | IDH1 c.395G>T (p.Arg132Leu)  IDH1 c.352C>T (p.Pro118Ser) | Astrocytoma, IDH-mutant, WHO grade II | Stereotactic needle biopsy  Radiotherapy (proton beam irradiation)  Temozolomide |
| **Patient 3** | IDH1 c.395G>A; p.(Arg132His) | Anaplastic astrocytoma, IDH-mutant, WHO grade III | Subtotal resection  Radiotherapy  Temozolomide  Lomustin |
| **Patient 4** | IDH1 c.395G>A; p.(Arg132His) | Astrocytoma, IDH-mutant, WHO grade II | Subtotal resection  Radiotherapy  Temozolomide |


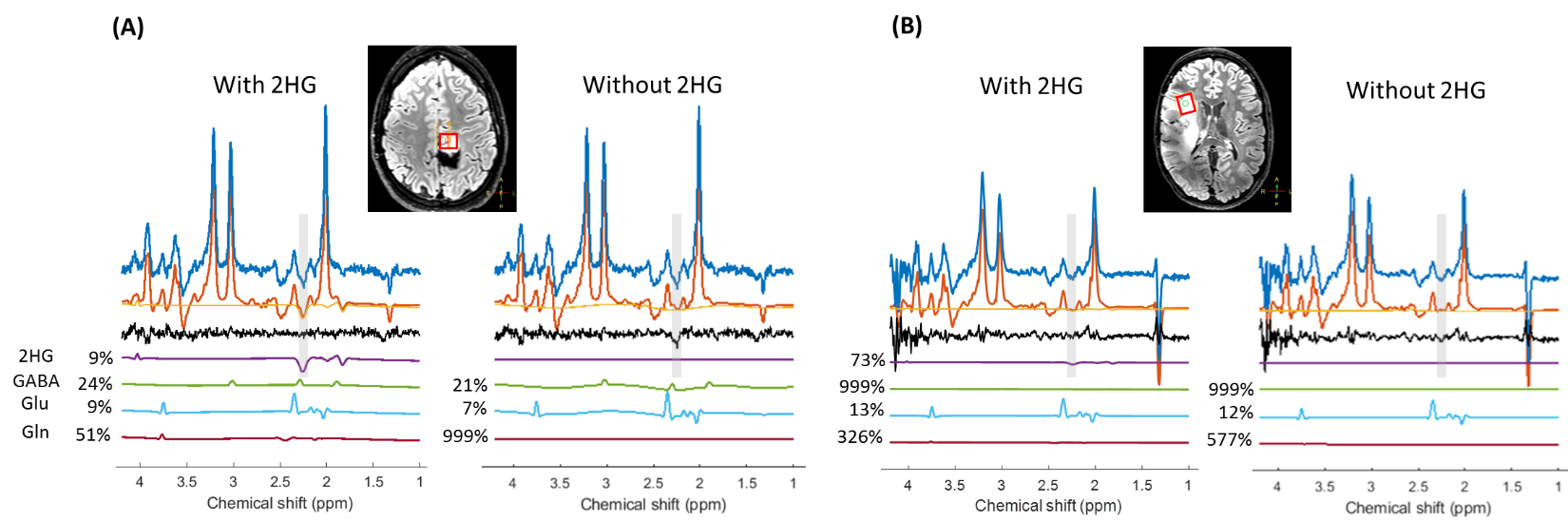


**Supplementary Figure 1.** sLASER spectra from patient 1 (A) and 3 (B) with LCModel outputs using basis sets with and without 2HG. Metabolite CRLBs are shown next to their fits.


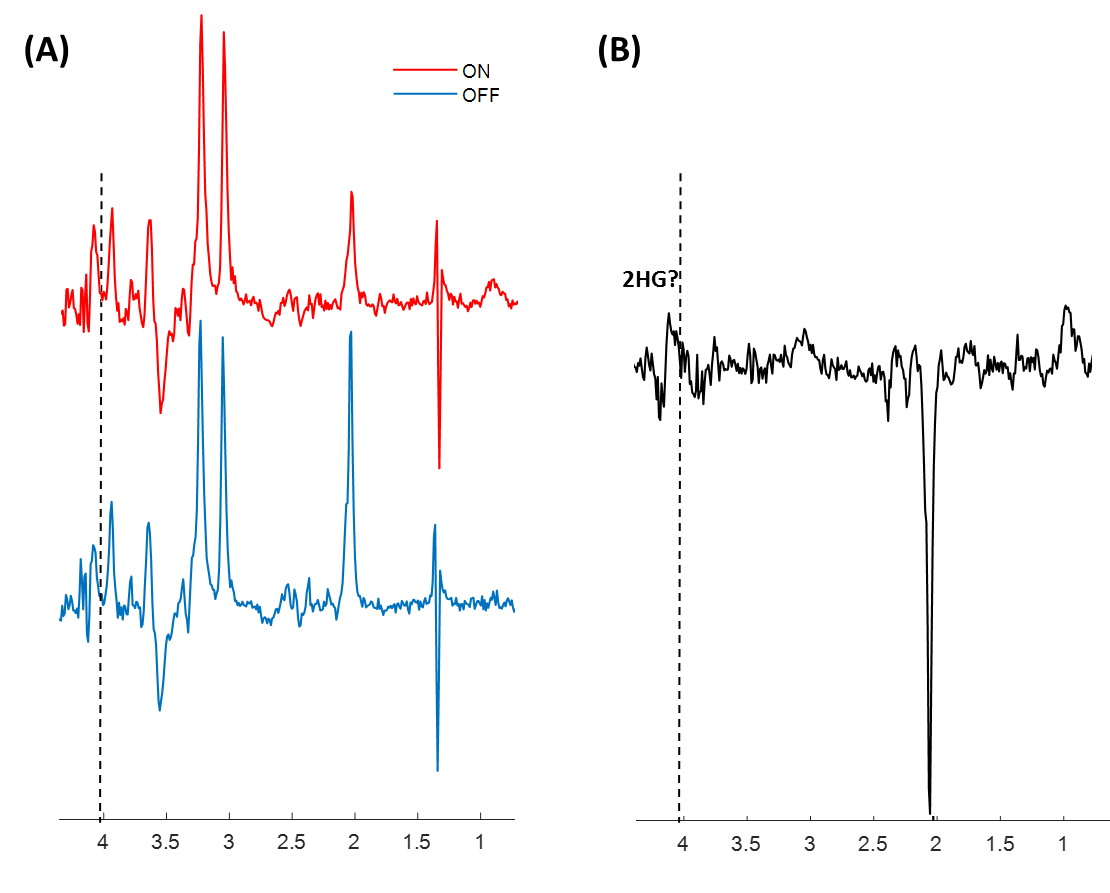


**Supplementary Figure 2.** An example of acquired spectra from MEGA-sLASER under suboptimal B_1_^+^ in the MRS voxel, leading to incomplete inversion during ON acquisition (A). In case of 2HG presence, the 2HG signal at 4.02 would not refocus completely. This may result in an ambiguous signal at that frequency especially when the 2HG level is too low. The subtracted spectrum (2 Hz apodization) is shown in (B).
